# The First Autoregulated Total Artificial Heart Implant in the United States

**DOI:** 10.1016/j.atssr.2022.09.007

**Published:** 2022-09-21

**Authors:** Jacob N. Schroder, Sharon L. McCartney, Piet Jansen, Ryan Plichta, Jason N. Katz, David M. Smadja, Krish C. Dewan, Carmelo A. Milano

**Affiliations:** 1 Department of Surgery, Duke University Medical Center, Durham, North Carolina; 2 Department of Anesthesia, Duke University Medical Center, Durham, North Carolina; 3 CARMAT SA; 4 Department of Cardiology, Duke University Medical Center, Durham, North Carolina; 5 Biosurgical Research Laboratory (Carpentier Foundation), Paris University and European Georges Pompidou Hospital, Paris, France

## Abstract

The Aeson total artificial heart provides right- and left-sided heart replacement for biventricular failure with notable improvements from prior generations. These include enhanced hemocompatibility and autoregulation enabling increased output in response to higher filling pressures. We report the first clinical implantation in the United States as part of an early feasibility study. The patient was successfully bridged to transplant after 5 months of support on the device and has made a full recovery.

There is a significant cohort of patients with biventricular failure who fail to establish adequate circulation with left ventricular assist device support alone. The Aeson (CARMAT SA) is the newest generation total artificial heart (TAH) that provides right- and left-sided heart replacement. This report describes the first clinical implantation in the United States as part of an early feasibility study. The inclusion criterion was severe biventricular dysfunction/failure assessed by echocardiographic and hemodynamic measurements. Patients with inadequate chest dimensions or advanced end-organ dysfunction are ineligible (https://clinicaltrials.gov/ct2/show/NCT04117295). The Aeson device has unique features relative to earlier TAH devices (Figure 1).^1^ Its blood-contacting surface consists of bovine pericardium, and it uses 4 bioprosthetic, bovine pericardial valves; this enhances hemocompatibility, reducing the need for anticoagulation.^2^ The device also features an electrohydraulically driven, pulsatile flow mechanism that mimics native ventricular contraction and seeks to reduce shear force applied to blood elements (Video). In addition, pump outputs are autoregulated on the basis of preload pressure changes measured inside the device.

**Figure 1:**
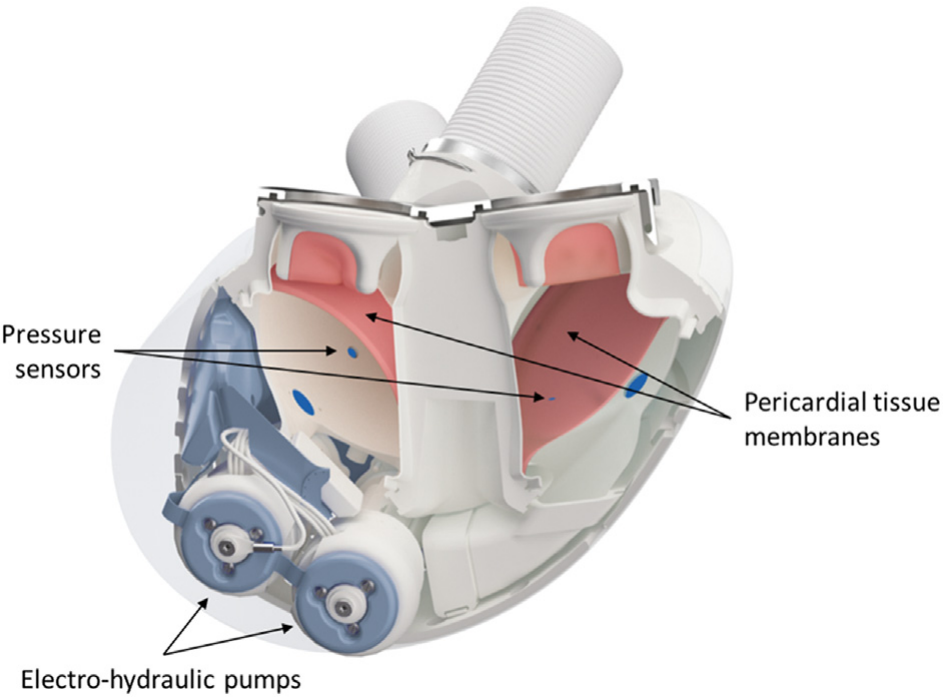
Diagram of Aeson (CARMAT) total artificial heart.

The patient is a 39-year-old man with diabetes and ischemic cardiomyopathy. He was hospitalized for increased symptoms of heart failure. Echocardiography demonstrated biventricular dysfunction with a left ventricular ejection fraction of 15%. While hospitalized, he experienced ventricular fibrillation requiring central venoarterial extracorporeal membrane oxygenation support. Venoarterial extracorporeal membrane oxygenation was converted to Impella 5.5 left ventricular assist device (Abiomed Inc). However, the patient continued to experience ventricular tachycardia and right-sided heart dysfunction. He was deemed to be transplant eligible, met inclusion criteria, and had no exclusion criteria for enrollment in the CARMAT early feasibility trial (https://clinicaltrials.gov/ct2/show/NCT04117295). Informed consent was obtained (IRB protocol # Pro00104138).

Preimplantation chest computed tomography scan showed a sternal–to–vertebral body distance of 173 mm (threshold, 126 mm) and a sufficient diaphragm–to–main pulmonary artery (PA) distance of 108 mm (Supplemental Figure 1). The operative steps and implantation technique are summarized in Supplemental Figure 2. After redo sternotomy and removal of the Impella device, cardiopulmonary bypass (CPB) was initiated with aortic-bicaval cannulation. After aortic cross-clamping, the aorta and main PA were transected as proximally as possible. Ventriculectomies were performed, preserving the mitral and tricuspid annuli with a small rim of ventricular tissue (3-5 mm). The annuli were separated from each other as much as possible. The coronary sinus was oversewn, and a percutaneous, transvenous catheter was placed across the interatrial septum for left atrial pressure monitoring. The left atrial appendage was clipped. Two prosthetic atrial cuffs lined with bovine pericardium were independently sewn to the annuli; an initial running suture apposed the cuff to the endocardium, avoiding any intervening muscle or epicardium. A second running suture was placed external to the first for hemostasis and incorporated the muscle rim. Next, a rectangular titanium atrial interface was fitted over and secured to the 2 sewing cuffs, and this rectangular metal interface was subsequently attached to the device. Dacron outflow grafts were cut to length and sewn to the main PA and aorta. The electrical power cord was tunneled out through the right rectus muscle. The device was filled and deaired with a vent placed in the ascending aorta. It was initially activated in a fixed mode, and the aortic cross-clamp was removed. Pump beat rate and stroke volume were gradually increased while the patient was weaned off CPB support (CPB time, 3 hours 52 minutes). Transesophageal echocardiography aided in deairing (Supplemental Figure 3). After hemostasis was achieved, the device was switched to autoregulation mode, in which beat rate increases in response to filling pressures, transduced within the pumping chambers (see Figure 1). Initial flows were 5 to 6 L/min with filling pressures around 10 mm Hg. Sternal wound closure was delayed for 24 hours. Total operative time was 7 hours 8 minutes.

Immediate postimplantation management required vasopressors to maintain arterial blood pressure and volume resuscitation. Pump flows stabilized at 6 to 6.5 L/min. Oliguria was treated with diuretics and brief venovenous hemofiltration. Renal, respiratory, and hepatic function gradually normalized. The pump was maintained in autoregulation, and operating typical parameters are displayed on the monitoring screen (Figure 2). During the first 30 days, there was only 1 alarm triggered by high inflow pressure, which correlated with the patient’s bearing down; it resolved spontaneously and was inconsequential.^3^^,^^4^ He was discharged home on postoperative day 75 in stable condition after a period of training. There were no postdischarge bleeding or thrombotic complications.

**Figure 2:**
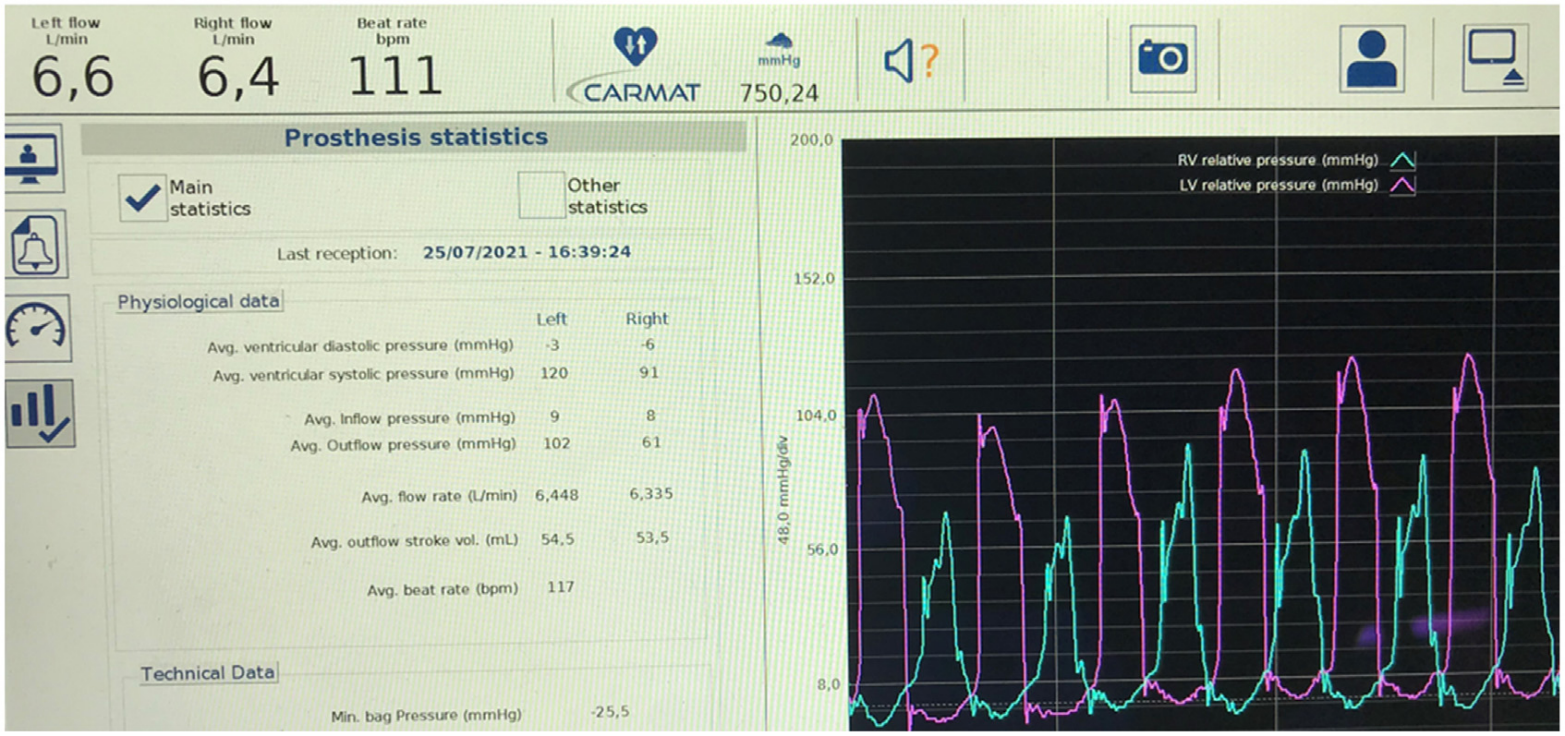
Main display screen showing continuous real-time right and left pump pressures, average flows, inflow/outflow pressures, and pressure in the hydraulic fluid bag.

Anticoagulation was delayed for 48 hours after implantation. Heparin was subsequently started intravenously and transitioned to daily low-molecular-weight heparin injections (enoxaparin, 150 mg daily) and daily aspirin (81 mg) on day 27. Normal platelet levels were seen (Figure 3A). We also studied high-molecular-weight multimers of von Willebrand factor (HMWM-VWF) before and after implantation, as described previously.^5^ At baseline, before Aeson implantation, a decrease in HMWM-VWF was observed that could be attributed to the existing Impella 5.5 support. At day 1 after Aeson implantation, HMWM-VWF increased significantly to normal levels and remained stable throughout support (Figures 3B, 3C).

**Figure 3:**
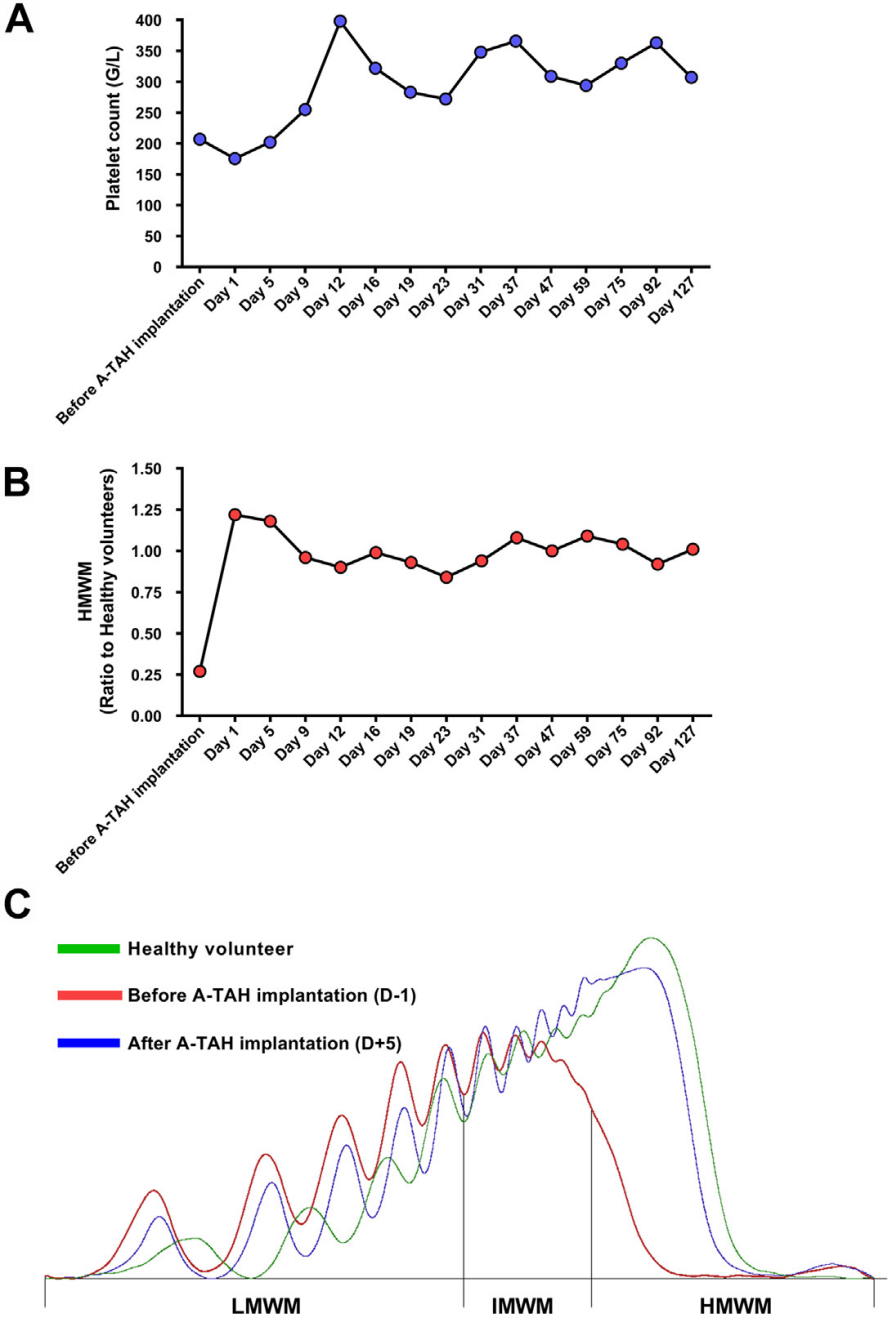
Hemocompatibility studies. (A) Platelet level before and after Aeson total artificial heart (A-TAH) implantation. (B) Quantitative analysis of different patterns of high-molecular-weight multimers (HMWM) of von Willebrand factor before and after implantation (reference standard [normal plasma] multimer ratio = 1). (C) Multimer densitometry in a healthy volunteer, during Impella implantation before A-TAH, and after A-TAH from low- (LMWM) to intermediate- (IMWM) and high- (HMWM) molecular-weight multimers.

After 5 months on support, a donor organ became available, and he underwent uncomplicated redo sternotomy, removal of the TAH, and cardiac transplantation. At 8 months after transplantation, there are no signs of allograft dysfunction or significant rejection.

## Comment

This first US experience demonstrates the effectiveness of the Aeson TAH. Most important, there were no bleeding complications, and the patient has been successfully bridged to a transplant after 8 months of continuous support. In addition, we confirmed prior studies showing that the device does not lead to degradation of von Willebrand multimers and resulting coagulopathy relative to rotary-flow ventricular assist devices.^2^ Given its greater hemocompatibility relative to current mechanical circulatory support, the Aeson could provide durable biventricular support with fewer strokes and bleeding-related adverse events. In addition, the ability to autoregulate based on sensed preload will better facilitate physical activity. Further assessment of its safety and performance will be provided by the ongoing US feasibility trial. Because of quality issues affecting some of the prostheses, the study sponsor temporarily suspended implants of this device in December 2021. Following the characterization of quality defects, corrective actions have been implemented within the production processes, with modified prostheses to be available in the fourth quarter of 2022. Food and Drug Administration authorization will then be requested to resume implants within the scope of the US early feasibility study.

## Supplementary Data

Video

Supplemental Figures1-3
