# Supplementary material for: The First Autoregulated Total Artificial Heart Implant in the United States
Source: Ann Thorac Surg Short Rep. 2022 Sep 21;1(1):185–7. doi: 10.1016/j.atssr.2022.09.007 (PMC11708379; doi:10.1016/j.atssr.2022.09.007)
Supplement: Supplemental Figures1-3 [file mmc2.pptx]

## Slide 1
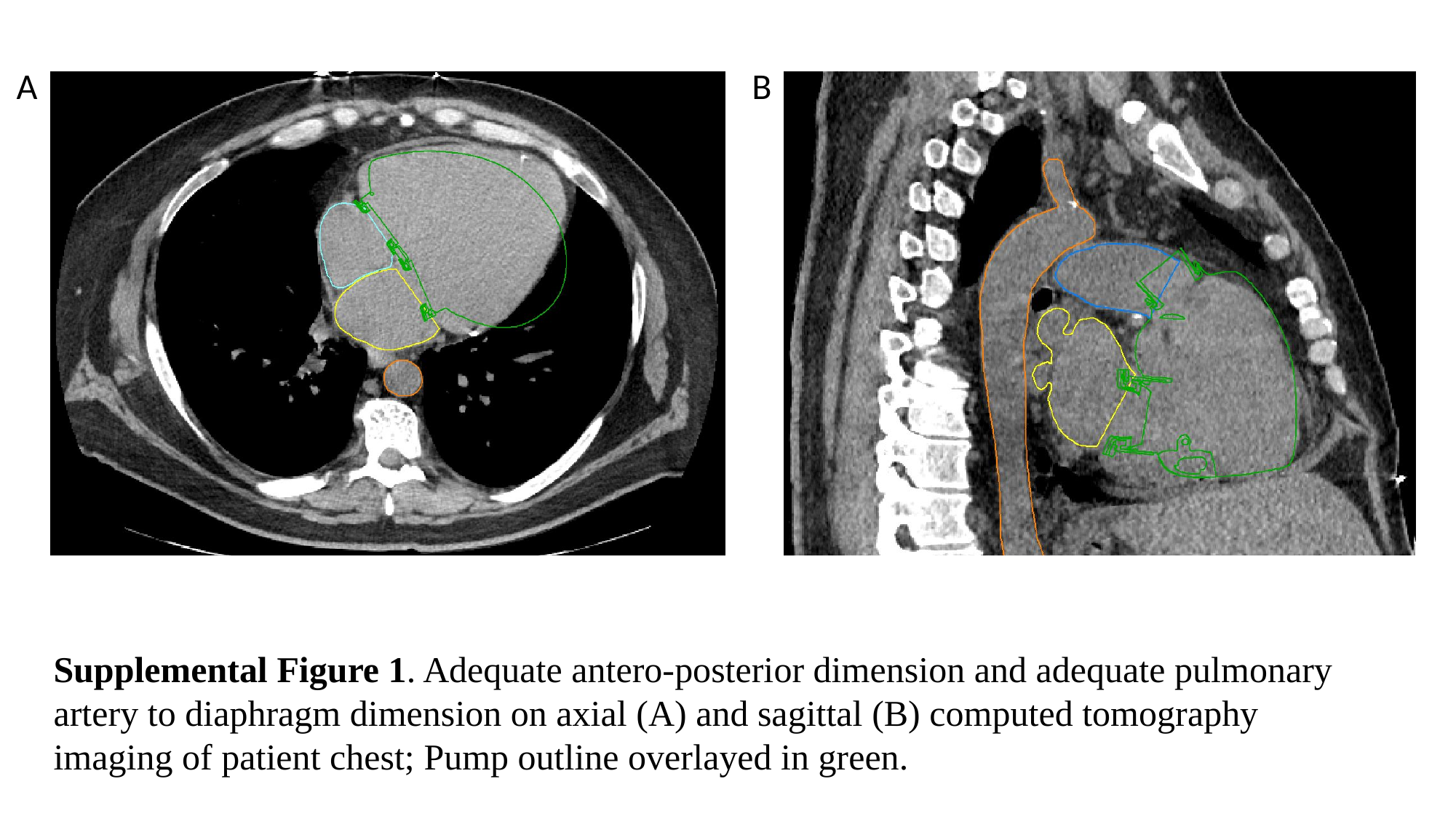

A
B
Supplemental Figure 1. Adequate antero-posterior dimension and adequate pulmonary artery to diaphragm dimension on axial (A) and sagittal (B) computed tomography imaging of patient chest; Pump outline overlayed in green.

## Slide 2
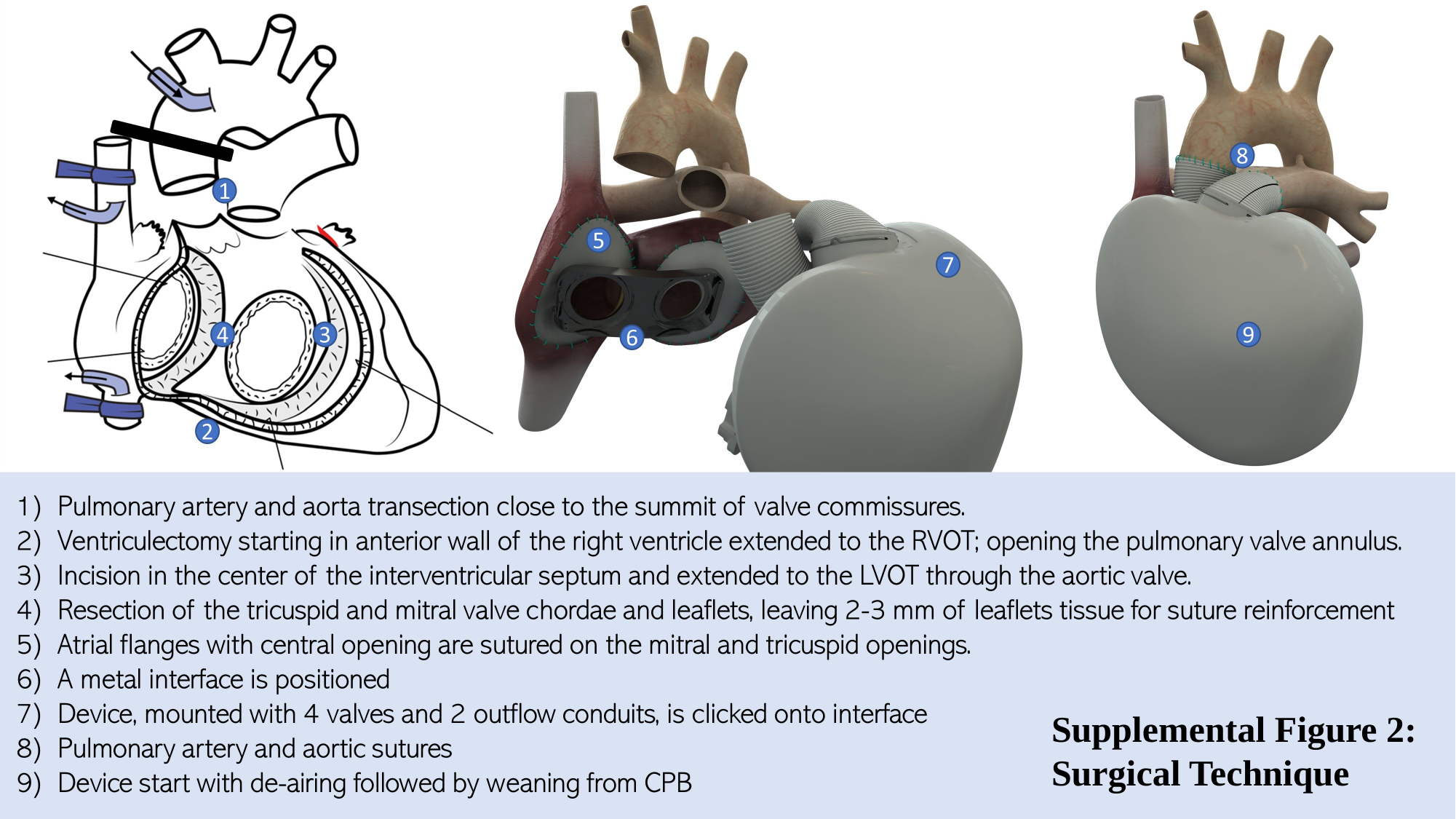

Supplemental Figure 2:
Surgical Technique

## Slide 3
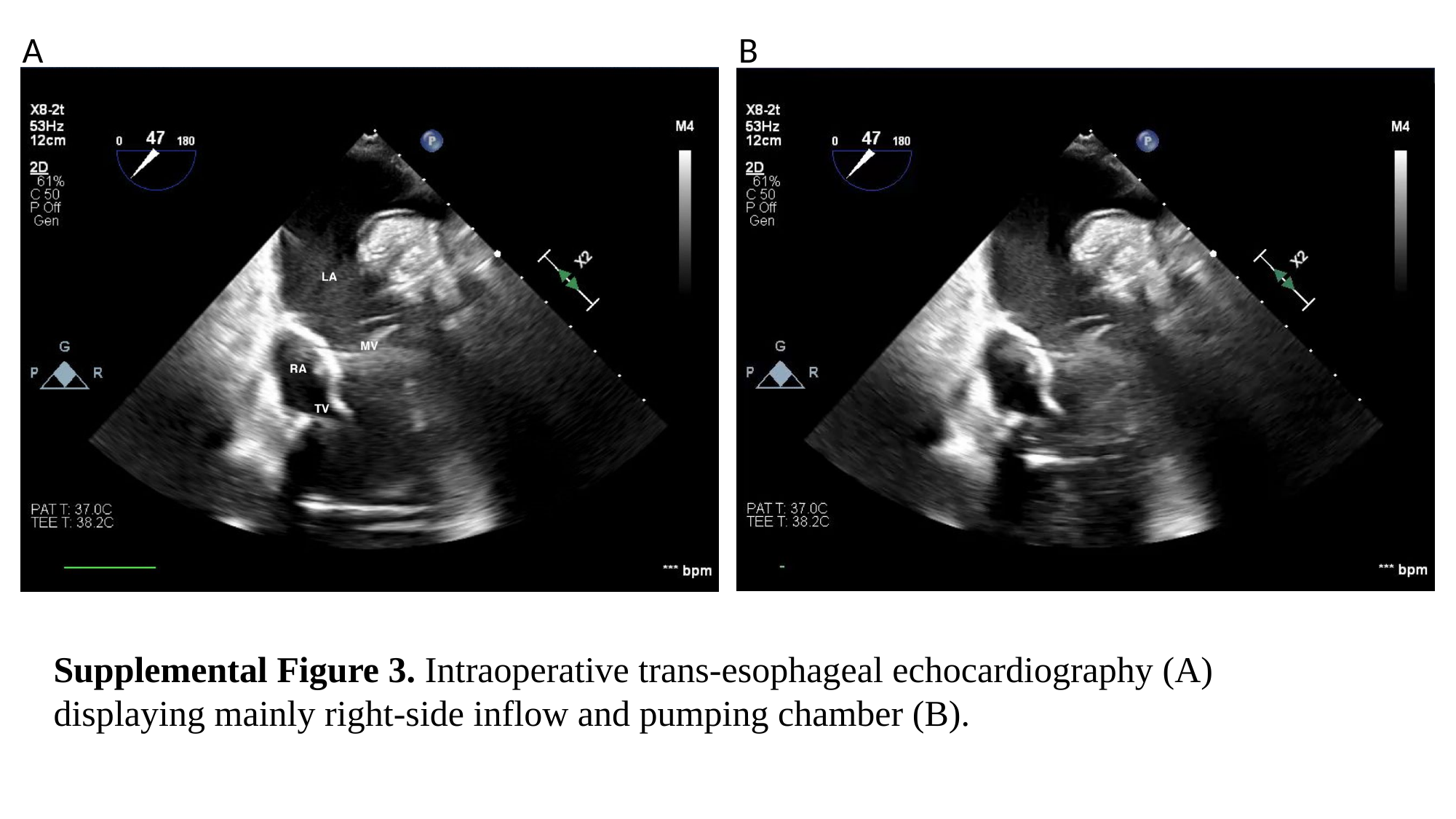

A
B
Supplemental Figure 3. Intraoperative trans-esophageal echocardiography (A) displaying mainly right-side inflow and pumping chamber (B).
